# Highly efficient and selective integrated directional couplers for multigas sensing applications

**DOI:** 10.1038/s41598-023-49889-2

**Published:** 2023-12-20

**Authors:** Ajmal Thottoli, Gabriele Biagi, Artem S. Vorobev, Marilena Giglio, Giovanni Magno, Liam O’Faolain, Marco Grande

**Affiliations:** 1 Department of Electrical and Information Engineering, Politecnico di Bari, 70126 Bari, Italy; 2 Centre for Advanced Photonics and Process Analysis, Munster Technological University, Cork, T12 T66T Ireland; 3 Tyndall National Institute, Cork, T12 PX46 Ireland; 4 PolySense Lab, Dipartimento Interateneo di Fisica, University and Politecnico of Bari, Via Amendola 173, 70126 Bari, Italy

**Keywords:** Integrated optics, Mid-infrared photonics, Optical sensors

## Abstract

The design and fabrication of a compact, low-loss, broadband directional coupler (DC) based duplexer operating in the near-infrared (NIR) region are demonstrated. The duplexer exhibits high selectivity and coupling efficiency (CE), for target wavelengths of 1530 nm and 1653.7 nm, making it applicable in systems for the multi-gas detection of ammonia and methane. The measured CE for the duplexer is 73% and 76% at 1530 nm and 1653.7 nm respectively. These results demonstrate the effectiveness of the duplexer as a broadband and scalable power source for highly sensitive sensing techniques, like quartz-enhanced photoacoustic spectroscopy (QEPAS). Its compact size and low-loss characteristics make it highly portable and well-suited for drone-based multi-gas detection applications.

## Introduction

Micro photonics has garnered significant attention in both spectroscopic and telecommunications applications, thanks to its enhanced processing speed, compact size, and the capability to integrate various optical functions onto a single chip^1,2^. In the field of telecommunications, wavelength division multiplexing (WDM) plays a pivotal role in optical signal manipulation for both upstream and downstream data transmission^3^. Analogous multiplexing techniques, as employed in optical communications, also find applicability in the domain of detection and analysis of multiple gases. In multi-gas sensing applications, a multitude of distinct wavelengths within the near-infrared (NIR) spectrum can be used to probe the physical and chemical properties inherent to the target gas molecules^4–6^. Various technologies are employed for gas sensing, including electrochemical, piezoelectric, thermal conductivity, mass spectrometry, and optical sensors^7–9^. One particularly promising technique is Quartz-Enhanced Photoacoustic Spectroscopy (QEPAS), which exploits a quartz tuning fork (QTF) for highly sensitive detection of trace gases in a small volume by enhancing weak photoacoustic signals. Absorption is determined indirectly by measuring the pressure wave created by local heating from the absorption of light, with the QTF acting as a resonant acoustic transducer^10–12^. Notably, QEPAS is a hyperspectral method applicable across a wide spectrum, spanning from visible light to the terahertz (THz) range. Multiple gases can be detected simultaneously by exciting different mechanical modes^13^.

A wavelength multiplexed light source thus promises to significantly expand the scope of such gas analyzers. Several optical passive components, including directional couplers (DC), Mach–Zehnder interferometers (MZI), arrayed waveguide gratings (AWG), and multimode interference couplers (MMI), facilitate multiplexing functionalities^14–17^. Among these options, the DC stands out as particularly promising due to advantages, such as a compact device footprint, high reliability, and the potential for large-scale production. Additionally, the DC offers numerous benefits, including low fabrication loss, minimal insertion loss, the capacity to handle multiple channels, and compatibility with other multiplexing devices^18,19^. These technologies enhance portability through integrated waveguide-based optical structures. They enable the miniaturization of bulk optical components and equipment while remaining compatible with optical sources and gas sensors. Ultimately, flip-chip integration or transfer printing of laser dies will provide a robust self-contained chip offering multiple wavelengths^20,21^.

**Figure 1:**
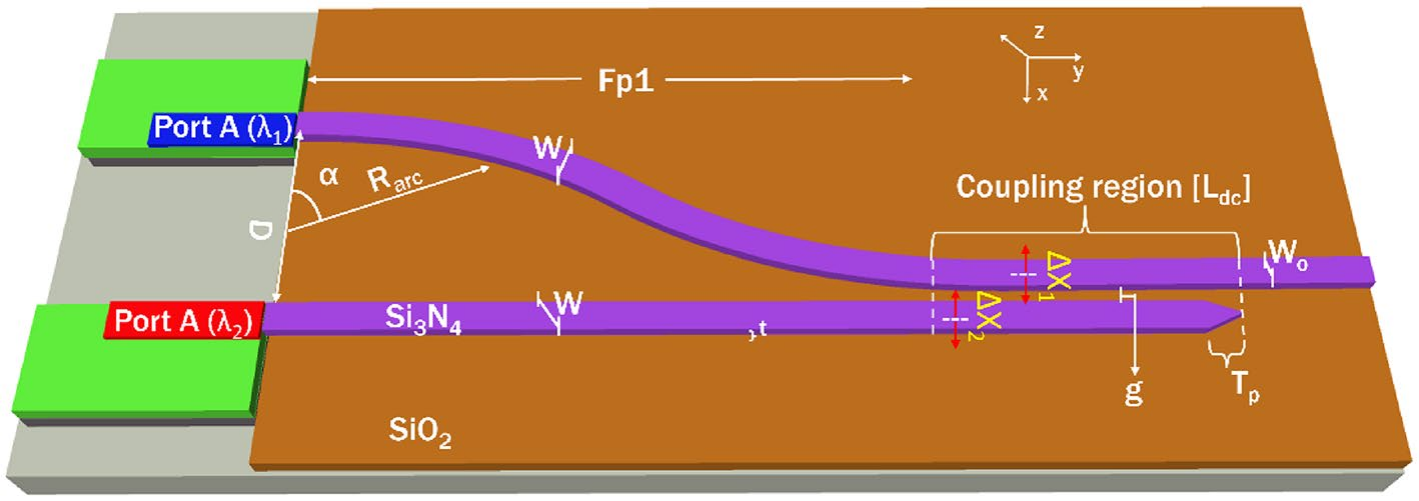
Schematic diagram of design parameters of directional coupler (DC) based wavelength duplexer. Port A refers to the bend waveguide (top) and Port B refers to the straight waveguide (bottom).

This paper focuses on the design, optimization, and characterizations of a low-loss, compact directional coupler-based duplexer. It explores design flexibility and coupling efficiency (CE) to facilitate the integration of spectral beams and their components on a single photonic chip, especially over a wide bandwidth, aiming miniaturized multi-gas sensing. Numerical investigations are conducted to determine the coupling efficiency (CE) for both the numerically calculated output beam profiles of commercially available diode lasers, including fundamental and higher-order mode profiles. The paper also examines strategies for filtering higher-order modes and safeguarding input sources against reflections returning to the source. The device is designed to effectively combine distinct wavelengths, namely *λ*_*1*_ = 1530 nm (Port A) and *λ*_*2*_ = 1653.7 nm (Port B). These wavelengths were chosen due to their suitability for multi-gas detection of ammonia and methane, as they exhibit distinctive optical absorption characteristics in the wavelength range covered by diode lasers. The numerical investigation provides a comprehensive evaluation of these design aspects. Figure 1 presents a schematic diagram of the envisaged directional coupler-based duplexer design with integrated lasers. The S-bend waveguide is used to guide *λ*_*1*_, as shorter wavelengths experience the least bend loss, while a straight waveguide is employed for guiding *λ*_*2*_, where the optical power available is typically lower. The parameter "*L*_*dc*_" represents the coupling length, denoting the distance along which two waveguides interact or couple with each other, and "*g*" represents the distance between the waveguides, indicating the spatial interval between them. The input waveguide ports are positioned *D* = 100 µm apart, and the bend radius in the design is set to *R*_*arc*_ = 5000 µm, with an angle *α* = 8.1°, half footprint Fp1 = 1400 µm, to ensure sufficient space for integrating the micro on-chip lasers and to minimize bend loss during light propagation. The optical power output is collected at the point *W*_*o*_.

## Design and optical intensity distribution

**Figure 2:**
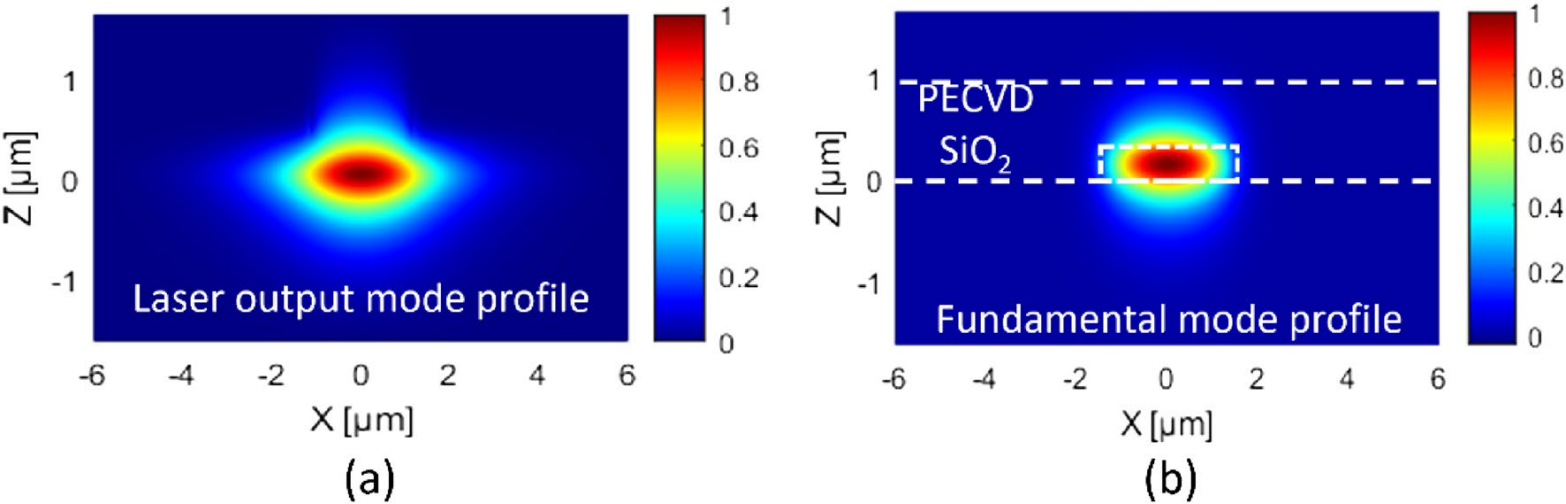
(**a**) Output beam profiles of numerically calculated commercially available (InP-based) diode laser. (**b**) Fundamental mode profile of the input waveguide at the *λ*_*1*_*.*

The dimensions of the input waveguide (W) of the coupler have been optimized to maximize transmittance while matching the output mode shapes of a commercially available diode laser, as shown in Fig. 2a. This highlights the motivation for utilizing the butt coupling technique to achieve effective laser-to-chip coupling. Extensive investigations were conducted on the waveguide width, thickness, cladding material (air, SiO_2_, PMMA and SU-8), and cladding thickness individually. The optimal configuration has been identified: *W* = 3 µm, thickness of the core equal to 300 nm, and cladding thickness with SiO_2_ material equal to 1 µm. Total output transmittance obtained for the bare input waveguide at wavelength *λ*_*1*_ and *λ*_*2*_ are 92% and 94%, respectively. These results provide valuable insights into the strategic design decision made for the input waveguide^22,23^. The simulations only consider the TE mode throughout the simulations, in anticipation of future diode laser integration that will operate in the TE mode.

The fundamental of the input waveguide for *λ*_*1*_ is shown in Fig. 2b, effective refractive index value of fundamental mode (M00) of the wavelength *λ*_*1*_ and *λ*_*2*_ are ${n}_{eff1,\lambda 1}$ = 1.644 and ${n}_{eff1,\lambda 2}$ = 1.620. Additionally, the input waveguide also houses higher order mode (M01), the effective indices are ${n}_{eff2,\lambda 1}$ = 1.518 and ${n}_{eff2,\lambda 2}$ = 1.476, for wavelength *λ*_*1*_ and *λ*_*2*_*,* respectively. The relation between the coupling length and optical transmittance was found using coupled mode theory, when the mode of each waveguide, in the absence of the other, remain nearly unchanged, and the coupling primarily modifies the amplitude of these modes without affecting their transverse spatial distribution and their propagation constant (β)^24^.

Here the waveguides are identical, and the phase mismatch Δ ${\beta }_{\lambda 1}=\Delta {\beta }_{\lambda 2}$ = 0. The length of evanescent mode coupling can be estimated as Lc = (π/(2К))·(2n + 1), where Lc is the coupling length, at a distance the power is completely coupled to the other waveguide, К the coupling coefficient and n (where n = 0, 1, 2…) is the number of beat length. The coupling coefficient depends on the gap between the waveguides and the effective refractive index of the waveguide. The calculated coupling coefficient for *λ*_*1*_ and *λ*_*2*_ nm are К_*a*_ = $4.245\cdot {10}^{-3}$ and К_*b*_ = $5.817\cdot {10}^{-3}$^25^.

**Figure 3:**
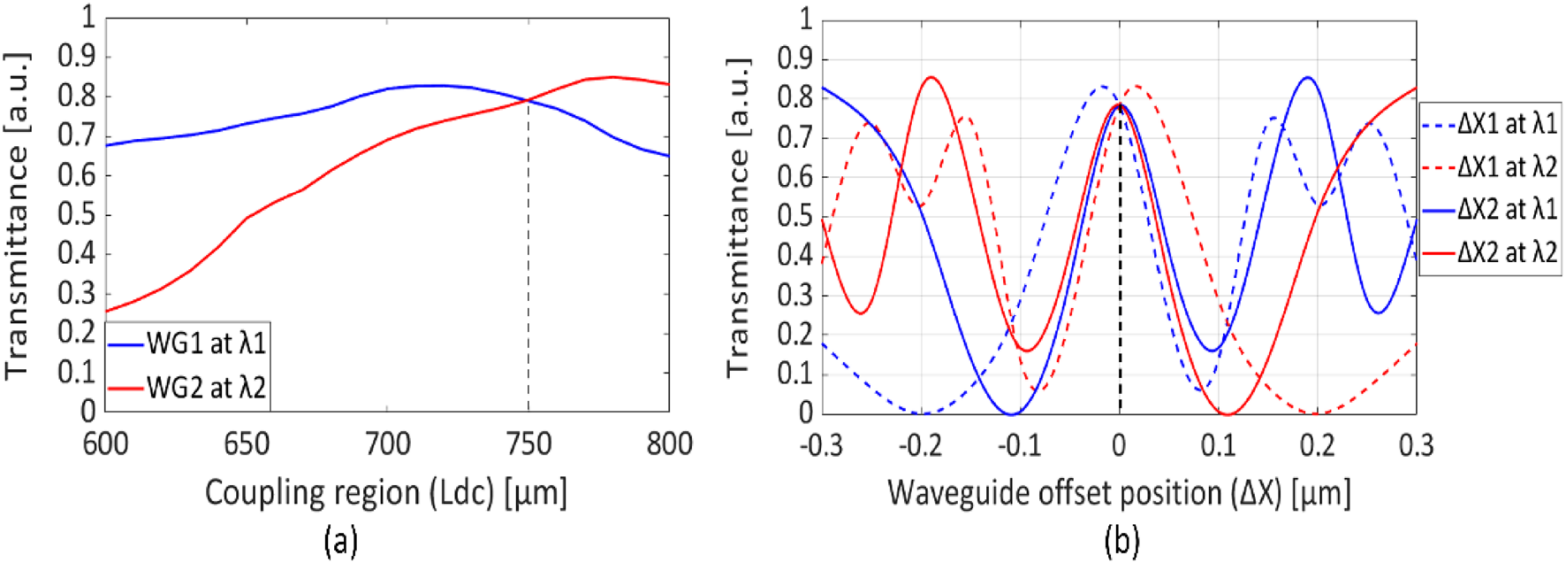
Transmittance of wavelength launched at port A and port B (**a**) with respect to the gap between the input waveguides and (**b**) with respect to length variation of port B.

Figure 3a depicts the transmittance (*T*_*o*_) determined for *L*_*dc*_ by launching laser mode profiles for *λ*_*1*_ and *λ*_*2*_ into respective input waveguide ports of the duplexer, while keeping the g constant at 300 nm. A relatively consistent response is observed for *T*_*o*_ over a range of several tens of micrometers of *L*_*dc*_ variations, ultimately, an *L*_*dc*_ value of 750 µm is chosen for the coupler. Furthermore, the sensitivity of *g* is investigated by varying the position of the input waveguide within a range of ± 300 nm. The optimal *ΔX* axis position is found to be at zero for launching *λ*_*1*_ and *λ*_*2*_, at respective input waveguides and changing the *ΔX*_*1*_ and *ΔX*_*2*_ alternatively, as shown in Fig. 3b. The design proves the position of input waveguides are highly sensitive, offering fluctuation in *T*_*o*_ values, with changes in the order of a few tens of nanometers. The duplexer shows a combined optical output power transmission of 79% at the output section *W*_*o*_ for wavelengths *λ*_*1*_ and *λ*_*2*_. This is determined by utilizing the computed laser mode profiles at *L*_*dc*_ = 750 µm and *g* = 300 nm.

**Figure 4:**
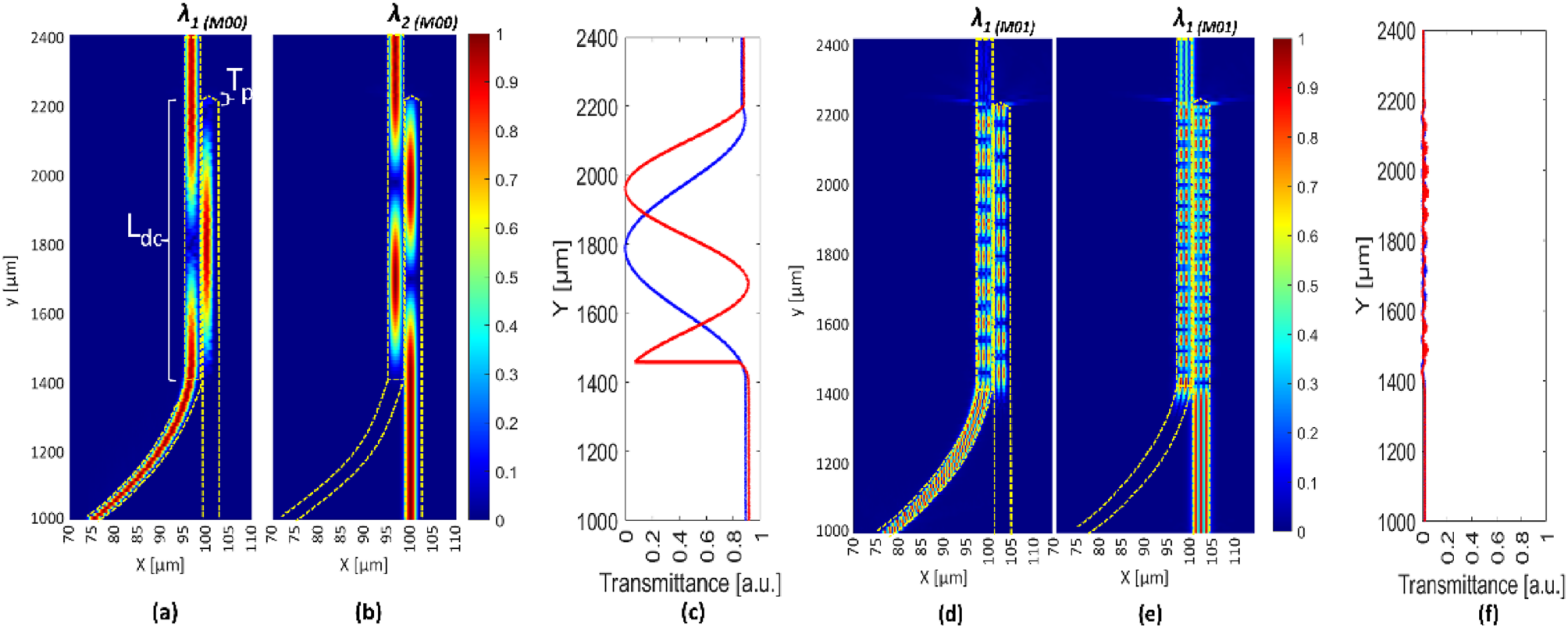
The electric field distributions of the DC coupler, when (**a**,**d**) Port A at 1530 nm and (**b**,**e**) Port B at 1653.7 nm are excited with the sole (**a**,**b**) fundamental mode or with the sole (**d**,**e**) higher order mode. (**c**,**d**) Transmittance fraction of the (**c**) sole fundamental mode and of the (**f**) sole higher order mode as a function of the y-coordinate, when the two ports are separately excited by launching the laser mode profile.

Higher-order modes can significantly impact waveguide transmittance by causing modal interference, and crosstalk^26^. To account the distribution of modes within the waveguide, both behaviors of fundamental mode and of higher order mode are investigated when launching the laser mode profile. In Fig. 4, the field distribution of both the fundamental mode and higher order mode launched at respective input ports are shown. Simultaneously, Fig. 4c illustrates the transmittance due to the sole fundamental mode launching the laser mode profile, showing transmittance values of 86% at 1530 nm and of 87% at 1653.7 nm. Similarly, Fig. 4d,e pertain to higher order modes launched at their respective ports, while Fig. 4f represents the transmittance of the sole higher order modes when launching laser mode profile. Notably, the transmittance of higher order modes is 0.1% and 0.15% for wavelengths of 1530 nm and 1653.7 nm, respectively. The total transmittance (*T*_*o*_) achieved by effective tapering at the terminal point of Port B is considered. The tapering length (*T*_*p*_) is optimized to 10 µm, considering the overall *T*_*o*_ of *λ*_*1*_ and *λ*_*2*_ using the laser mode profile, and with higher-order modes. Minimizing the effect of higher-order modes can lead to a higher signal-to-noise ratio in spectroscopic systems, showcasing the design's efficiency in transmitting optical power for specified wavelengths. Furthermore, the waveguide tapering also serves the purpose of reducing reflections from propagating back to the input source when interacting with slab SiO_2_ waveguides.

**Figure 5:**
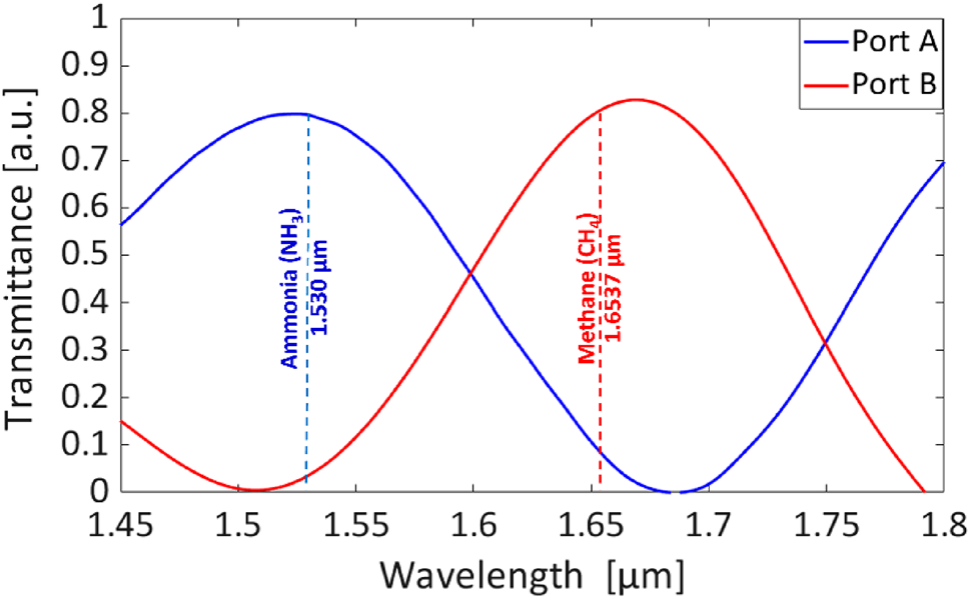
Range of wavelength launched at Port A and Port B.

The transmittance characteristics for a wavelength range 1450–1800 nm launched at Port A (blue lines) and Port B (red lines) are presented in Fig. 5. Also, the designed duplexer exhibits an optimal performance within the spectral range of 50 nm centered around the target wavelength (dashed vertical lines). The dimension of the waveguide remains constant throughout propagation in the output waveguide, thereby preserving the guiding mode and mode confinement.

## Experimental results

**Figure 6:**
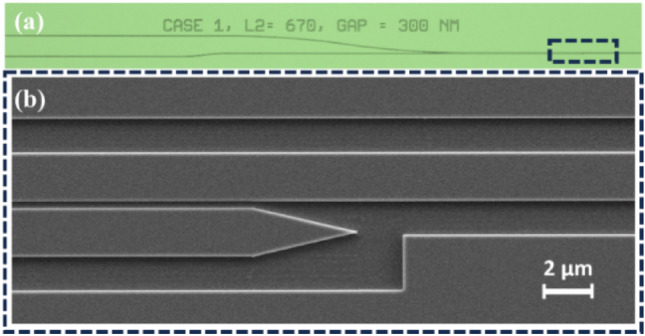
(**a**) Microscopic image of fabricated duplexer. (**b**) Details SEM imaging showing the tapered tip of the waveguide (dashed rectangle box in zoom).

Figure 6 shows the details of the fabricated DC duplexer, involving tapered tip of the waveguide corresponding Port B, employed to reduce any kind of reflections which could destabilize the laser*.* To normalize the device performance, a straight waveguide is included in the fabricated chip, having the same width (*W* = 3 µm) and thickness (*t* = 300 nm) as the designed duplexer (4 mm footprint). The input waveguides of each duplexer device were separated by *D* = 100 µm using S-bend waveguides (*R*_*arc*_ = 5000 µm, with an angle *α* = 8.1°, see Fig. 1) providing sufficient space for bonding the diode laser to the chip.

**Figure 7:**
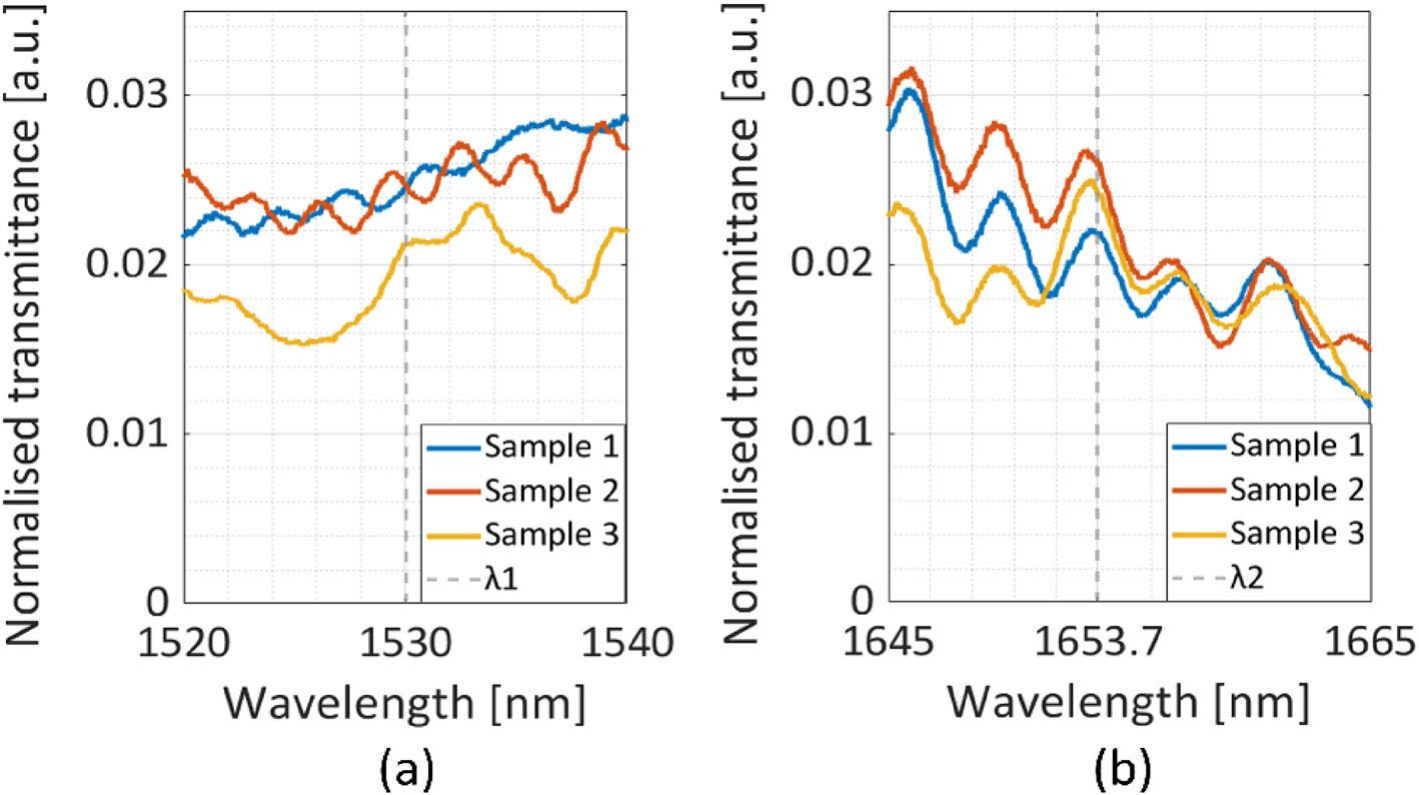
Experimental results for launching a range wavelength at waveguide corresponds to (**a**) *λ*_*1*_ and (**b**) *λ*_*2*_.

Individual alignment procedures were conducted for each wavelength due to the wavelength dependence of the optics employed during the characterization process. The measurement results of the best devices are shown in Fig. 7. The obtained transmittance spectra are normalized relative to the broadband laser source spectrum. The vertical line in the figures indicates the target wavelengths. The CE calculation procedure includes measures to address the signal-to-noise ratio and source spectra. This involves computing the transmittance of target wavelengths while normalizing them relative to the straight waveguide. The employed normalization processes are used the output spectra of the sources under the same conditions as those applied to the chip. Further, setting the maximum transmittance at the target wavelengths with respect to the source. Subsequently, Table 1 displays the calculated CE of each sample, referencing it to the established maximum transmittance.

**Table 1:** The coupling efficiency of the duplexer configurations.

Sample	L_dc_ [µm]	g [nm]	CE at λ_1_ [%]	CE at λ_2_ [%]
1	670	250	72.5	64.6
2	670	300	73	77
3	770	150	71.8	62

This normalization process facilitates accurate and meaningful comparisons across different samples and experimental conditions. These findings indicate that the designed device exhibits an optimal performance within a spectral range of approximately 10 nm centered around these specific wavelengths.

The presented characterization results of the fabricated DC device prove its consistent performance and reliability. Sample 2 demonstrates the most promising performance, achieving coupling efficiencies (CE) of 73% and 77% for *λ*_*1*_ and *λ*_*2*_, respectively, with *L*_*dc*_ = 670 µm and g = 300 nm. The measured *L*_*dc*_ shows an approximate 80 µm deviation from the predicted values in simulations, characterized by a flat response within that region. Sample 1 and Sample 2 both demonstrate strong performance, which can be attributed to a trade-off in CE values. These strong performances are driven by fluctuations in the g-value (see Fig. 3b), resulting in a highly variable CE response. These results provide a crucial optical component in on-chip devices, functioning as a low loss combiner with a footprint of 2 mm × 0.3 mm. The optical losses observed in the measurement can be attributed to various experimental factors. These factors include difficulties in effectively coupling the light to the input port of the chip, which involves employing a free-space end-fire setup with lenses to resize the optical fiber mode to match the waveguide mode. The characterized waveguide in this setup demonstrates a propagation loss of – 4 dB/cm. Additionally, imperfections in the fabrication process, as well as parasitic reflections within the waveguide structure caused by cleaved facets, contribute to the losses. Furthermore, the filtering of higher-order modes within the tapered waveguide region also plays a role in the overall reported losses. Table 2 displays the performance comparison with wavelength multiplexers previously reported^27–31^.

**Table 2:** Performance comparison with previously reported wavelength multiplexers.

References	Structure	Wavelength [nm]	Method	Insertion loss [dB]
^13^	DC	1310–1550	Simulation	0.27 and 0.08
^15^	MMI	1550–2000	Experimental	0.14 and 1.2
^26^	SWG DC	1550–2000	Simulation	0.14 and 0.80
^27^	TMI	1310–1550	Experimental	1
^28^	SWG MMI	1310–1550	Simulation	< 0.24
^29^	SWG MMI	1310–1550	Simulation	0.09 and 0.08
^30^	ADC	1310–1490–1550	Experimental	0.98, 0.69 and 0.76
-	This work	1530–1653.7	Experimental	1.37 and 1.13

## Conclusion

A compact directional coupler structure-based duplexer has been investigated, provided with the higher output power coupling ratio for combining wavelengths equal to 1530 nm and 1653.7 nm. The coupling efficiencies have been thoroughly examined and validated for various mode profiles, including the fundamental, higher-order modes and commercially available diode laser mode profiles. The numerically evaluated coupling efficiency for commercially available laser modes is 79% for wavelengths of 1530 nm and 1653.7 nm, respectively. However, the measured coupling efficiency reaches up to 73% and 77% for the same wavelengths, 1530 nm and 1653.7 nm, respectively. This increased coupling efficiency will greatly enhance the sensitivity and the selectivity for the detection of ammonia and methane gases, respectively. In fact, rather than using separate systems of detectors for each gas of interest, an on-chip laser combined with sensors can be used with the proposed duplexer to detect multiple gases.

## Methods

### Simulation method

3-dimensional simulations were performed using the Beam PROP simulation tool from RSoft. This platform is commonly used for parameterizing and estimating the optimum results using Finite Differences Beam Propagation Method (FD-BPM). Through the utilization of a convergence test, the mesh grid resolution has been optimized, resulting X grid = 50 nm, Y grid = 50 nm, and Z grid = 100 nm. The simulation involves a full duplexer design, including S-bends to account for coupling in the transition between bend and straight waveguide regions. Fundamental and higher-order mode properties, as well as the effective refractive index, were determined using the same tool. For the analysis of optical material properties, separate dispersion relations were applied to both silicon nitride and silicon dioxide materials using the Sellmeier equation.

### Device fabrication

The duplexers were fabricated using the standard CMOS compatible method^32,33^. To optimize fabrication tolerance and transmittance, various device configurations are employed. Initially, a 450 nm thick layer of ZEP 520A resist was spin-coated onto a 300 nm thick layer of Si_3_N_4_, deposited using plasma-enhanced chemical vapor deposition (PECVD), on a thermally oxidized 4-inch bulk silicon wafer (with a box thickness of 2.2 µm). Then the devices layouts were patterned on the resist by means of electron beam lithography (EBL) using 100 kV voltage and later developed in a bath of n-Amyl Acetate solution for 90 s and rinsed with IPA. The patterns were then transferred to the PECVD Si_3_N_4_ layer through inductively coupled plasma (ICP) etch step in O_2_:CHF_3_ chemistry in a 4:21 ratio (etch rate of ∼ 90 nm/min). The residual resist was later removed through a bath in 1165 Remover for 30 min and an O_2_ plasma ashing step. After the SEM inspection, the 1 µm-thick SiO_2_ layer was deposited with PECVD. A trench possessing a width of 2 µm is made in preventing the waveguide mode from escaping into the adjacent area.

### Characterization setup

The optical characterization of the fabricated sample was performed using an end-fire measurement set up^33^. Individual alignment procedures for each wavelength are carried out, using the Yenista Optics TLS, for Port A at 1530 nm, and a diode laser from Eblana Photonics for Port B at 1653.7 nm. The light generated is fed to an aspheric lens (10 ×) through optical fiber and a polarizing beam-splitter (PBS) is used to filter out the TM components. At this stage, the light is coupled to the device using a 60 × lens. A symmetrical arrangement is carried out to collect output. The alignment is achieved by maximizing the output power, derived from *W*_*o*_, using a Thorlabs power meter. Following the alignment process, the TLS is replaced with an Amonics ALS broadband light source, while the diode laser is substituted with an Amonics ASLD super luminescent source. The resulting *W*_*o*_ transmittance spectrum for each port is separately collected using a Yenista Optics Optical Spectrum Analyzer (OSA).

## Data Availability

Data implicit in the results submitted in this report are not publicly accessible at this time but may be obtained from the corresponding author upon reasonable request.
